# *Musa Paradisiaca* derived intrinsically heteroatom doped carbon dots as antioxidant and controlled drug release behavior

**DOI:** 10.1371/journal.pone.0329116

**Published:** 2025-08-14

**Authors:** Samiah A. Alhabardi, Jawza A. Almutairi, Gadah A. Al-Hamoud, Zainab Zaki Zakaraya, Umme Hani, Zahrah Ali Ahmed Asiri, Islam Al Freahat, Walaa Alsafadi, Wael Abu Dayyih, Sharuk L. Khan, Md. Zamshed Alam Begh

**Affiliations:** 1 Department of Pharmaceutics, College of Pharmacy, King Saud University, Riyadh, Saudi Arabia; 2 Department of Pharmaceutical Science, College of Pharmacy, Princess Nourah Bint Abdulrahman University, Riyadh, Saudi Arabia; 3 Department of Pharmacognosy, College of Pharmacy, King Saud University, Riyadh, Saudi Arabia; 4 Faculty of Pharmacy, Al-Ahliyya Amman University, Amman, Jordan; 5 Department of Pharmaceutics, College of Pharmacy, King Khalid University (KKU), Abha, Saudi Arabia; 6 Department of Biopharmaceutics & Clinical Pharmacy, Faculty of Pharmacy, Al-Ahliyya Amman University, Amman, Jordan; 7 Department of Pharmaceutics and Pharmaceutical Technology, Faculty of Pharmacy, Alahliyya Amman University, Amman, Jordan; 8 Faculty of Pharmacy, Mutah University, Al-Karak, Jordan; 9 Department of Pharmaceutical Chemistry, N.B.S Institute of Pharmacy, Ausa, Latur, Maharashtra, India; 10 Department of Pharmacy, Faculty of Allied Health Sciences, Daffodil International University, Dhaka, Bangladesh; Institute of Biophysics Chinese Academy of Sciences, CHINA

## Abstract

This work presents a green, single-step hydrothermal synthesis of intrinsically nitrogen-doped Carbon dots (MCDs) derived from Musa paradisiaca, a low-cost and renewable biomass source. The eco-friendly synthesis avoids external dopants or harsh chemicals, offering a scalable and sustainable alternative to conventional multistep methods. The resulting MCDs, with an average particle size of 4.2 nm (TEM), display desirable surface functionalities (FTIR, XPS) and heteroatom doping. Optical characterization revealed a broad UV-vis absorption at 280 nm and strong blue photoluminescence at 440 nm. DLS and zeta potential measurements confirmed excellent colloidal stability. The MCDs demonstrated high antioxidant activity (>80% radical scavenging) and biocompatibility in cellular assays. Moreover, they enabled controlled drug release, underlining their promise as multifunctional nanocarriers. Given their green synthesis, stability, and performance, these MCDs are highly suitable for future biomedical and clinical applications, particularly in antioxidant therapy and targeted drug delivery.

## 1. Introduction

Carbon dots (CDs) are fluorescent carbon nanomaterials measuring less than 20 nm, characterized by significant chemical stability, brilliant cytocompatibility, and good polarity, making them extensively utilized in diverse biomedical applications [1]. Initially identified inadvertently throughout the refinement of single-walled carbon nanotubes, they can now be promptly synthesized from several materials using multiple efficient strategies, including top-down approaches, microwave-assisted fabrication techniques, hydrothermal/solvothermal reaction, electrochemical exfoliation, and liquid-phase exfoliation [2,3]. CDs are generally classified as CDs, graphene quantum dots (GQDs), and polymer dots [4]. Hydrothermal synthesis is a prevalent technique for the production of CDs. In the hydrothermal synthesis process, dehydrating agents and/or oxidizing agents are commonly utilized, including H_2_O_2_ [5,6], H_2_SO_4_ [7,8], phosphorus pentoxide [9], and phosphoric acid [10]. Microwaves have likewise been employed for CD synthesis [11–14]. The properties of CDs are partially contingent upon the initial raw materials utilized in their hydrothermal synthesis. The raw materials may include sugars [15,16], amino acids [17,18], organic molecules [19,20], food waste [21], coffee [22,23], honey [24], juice [25], proteins [26], milk [27], and plastic bags [28,29]. The synthesized CDs derived from these templates exhibited unique photophysical and biological characteristics. Identifying an alternative ecologically sustainable method for synthesizing highly luminous CDs with novel characteristics appropriate for biomedical applications is essential [30]. Fluorescent CDs measuring less than 10 nm, exhibiting exceptional biocompatibility, minimal to negligible cytotoxicity, high quantum yield, non-blinking properties, and affordability, are seen as a promising advancement in nanomedicine [31]. Nonetheless, regarding emission tenability, the regulation of CDs’ emission remains under development as comprehension of the interaction between surface states and intrinsic states in photoluminescence (PL) advances. Recent progress in nanomaterial design has led to the development of highly luminescent CDs with tunable emission colors, including bright green- and red-emitting variants, which show exceptional promise for biological imaging applications. These fluorescent nanostructures combine several advantageous properties for biomedical use, such as stable light emission, low toxicity, and good water solubility. However, while their imaging capabilities have been extensively explored, their potential as traceable drug carriers remains significantly underdeveloped, with only a handful of studies addressing this important application. The rich surface chemistry of CDs allows for direct drug attachment through various reactive groups, while their inherent optical properties enable visualization of drug transport pathways in biological systems. This dual functionality suggests exciting possibilities for creating combined therapeutic and diagnostic platforms. However, critical questions remain regarding the efficiency of drug conjugation, the stability of these complexes in physiological environments, and the reliability of using the fluorescence signal to monitor drug distribution. Addressing these challenges could unlock new opportunities for developing smarter, traceable drug delivery systems with built-in monitoring capabilities [32]. Nevertheless, there are only a limited number of studies demonstrating the application of CDs as a drug delivery device, as the majority of research has concentrated on their optical features [33]. There has been considerable progress in the application of CDs for drug anchoring. The occurrence of medication-induced toxicity to healthy cells due to non-specific drug delivery is extensively recognized [34]. Their intense PL enables their application in cellular imaging. The MCDs serve as a delivery mechanism for doxorubicin (DOX) in cell imaging and cancer treatment. Yet a viable option to alleviate this problem is the implementation of the CDs delivery pathways. Jia et al. utilized polyethylene glycol (PEG) modified CDs to enhance the directed delivery of medicines to the tumor microenvironment [35]. This method significantly reduced medication leakage under physiological settings and alleviated the negative effects related to the treatment. As a result, the anticancer activity of DOX was increased [36]. Although various studies have concentrated on the fabrication and surface engineering of CDs, there is a paucity of literature concerning their drug transport potential [37]. This paper introduces an innovative technique for synthesizing intrinsically heteroatom-doped CDs from Musa paradisiaca (banana) peels, a sustainable and cost-effective natural resource. The environmentally conscious synthesis method ensures a sustainable process, aligned with the principles of green chemistry. The resultant CDs exhibit inherent doping with nitrogen and sulfur, sourced directly from natural precursors, hence obviating the need for additional chemical dopants. The inherent doping of heteroatoms markedly improves the PL characteristics, antioxidant efficacy, and biocompatibility of the CDs [38,39]. These CDs exhibit significant antioxidant activity due to the combined effects of their nanoscale dimensions, surface functions, and heteroatom doping, rendering them highly effective in neutralizing ROS. The CDs were evaluated for controlled drug release using a model drug, exhibiting pH-responsive behavior by physiological conditions. The customizable medication release feature highlights their potential as an intelligent nanovehicle for drug delivery applications. Investigations into cytotoxicity on human cell lines confirmed the non-toxic characteristics of the CDs, ensuring their appropriateness for biomedical applications. This study is novel due to its incorporation of sustainable synthesis, intrinsic heteroatom doping, and dual functionality as antioxidants and drug delivery agents. This study used a readily available agricultural waste product to address environmental challenges while presenting a multifunctional nanomaterial with considerable potential for biomedical applications. This study offers a benchmark for the development of cost-effective, environmentally friendly, and multifunctional nanomaterials sourced from natural resources.

## 2. Experimental section

### 2.1 Materials and methods

The banana (*Musa paradisiaca*) for CDs synthesis was brought from a local supermarket (Mumbai, India). 2,2′-azinobis (3-ethylbenzothiazoline-6-sulfonic acid) (ABTS) and 2,2-diphenyl-1-picrylhydrazyl (DPPH) were supplied by Sigma-Aldrich (Germany). Sodium hydroxide (NaOH), hydrochloric acid (HCl), sodium chloride (NaCl), sulfuric acid (H_2_SO_4_), quinine sulfate (QS), and 3-(4,5-dimethylthiazol-2-yl)-2,5-diphenyltetrazolium bromide (MTT) were acquired from SigmaAldrich. Dimethyl sulfoxide (DMSO) was acquired from Himedia. DOX was obtained from TCI Company. All studies were conducted using Milli-Q water.

#### 2.1.1 *Synthesis of MCDs.*

MCDs were manufactured from banana peels (*Musa paradisiaca*) by an eco-friendly hydrothermal technique. Fresh banana peels were gathered, meticulously cleansed using deionized water to eliminate surface contaminants, and then dehydrated at 60°C until entirely devoid of moisture. The desiccated peels were then pulverized into a fine powder using a mechanical grinder. A precise amount of banana peel powder (~10 g) was suspended in 100 mL of deionized water and thereafter placed into a Teflon-lined stainless-steel autoclave. The reaction occurred at 180°C for 8 hours under autogenous pressure. Upon cooling to ambient temperature, the resultant brown dispersion was subjected to filtration through a 0.22 µm membrane to eliminate bigger particles and insoluble residues. The filtrate was then centrifuged at 10,000 rpm for 10 minutes to further the purification of the MCDs. The supernatant containing the CDs was subjected to dialysis against deionized water using a dialysis membrane (molecular weight cutoff: 1 kDa) for 24 hours to remove small compounds and unreacted precursors. The cleaned MCDs were gathered, freeze-dried, and preserved in an airtight container for future research and usage. This approach produced extremely fluorescent, water-dispersible CDs with intrinsic heteroatom doping sourced from the natural composition of banana peels, therefore obviating the need for external doping agents. The synthesized MCDs were examined to verify their structural, optical, and functional features, confirming their appropriateness for antioxidant activity and controlled drug release investigations.

#### 2.1.2 *Synthesis of MCDs-drug conjugates.*

MCDs were coupled with DOX to produce MCDs-DOX conjugates. Initially, purified MCDs were dissolved in deionized water, and the pH was adjusted to 8.5 using a 0.1 M NaOH solution to facilitate the activation of their surface carboxyl groups. 50 mg of DOX was dissolved in DMSO and added gradually to the MCD solution while maintaining continual stirring. The carboxyl groups of the MCDs were activated for conjugation using N-(3-dimethylaminopropyl)-N′-ethylcarbodiimide hydrochloride (EDC) and N-hydroxysuccinimide (NHS) in a 1:1 molar ratio. The EDC (20 mg) and NHS (12 mg) were included in the MCD-DOX mixture, and the reaction was agitated at ambient temperature for 24 hours in a nitrogen environment to reduce DOX degradation. Upon the end of the reaction, the MCDs-DOX conjugates were filtered to exclude unreacted DOX and byproducts. This was accomplished by dialysis with deionized water using a 2 kDa molecular weight cut-off dialysis membrane for 48 hours, accompanied by regular water exchanges. The dialyzed product was then freeze-dried to get the final MCDs-DOX conjugates in powdered form. The conjugation of DOX to MCDs was confirmed using UV-Vis spectroscopy, which revealed distinct absorption peaks for both DOX and MCDs, while FTIR analysis indicated the existence of amide bonds. The drug loading efficiency was determined by quantifying the unbound DOX in the dialysate by fluorescence spectroscopy. The MCDs-DOX conjugates exhibited superior water dispersibility, prolonged fluorescence, and pH-responsive drug release characteristics, making them appropriate for targeted drug delivery applications.

#### 2.1.3 *Drug release study.*

The drug release behavior of MCDs-DOX conjugates was evaluated under simulated physiological conditions, using phosphate-buffered saline (PBS) at different pH values (pH 7.4 and pH 5.5) to mimic the normal and acidic environments of the human body, respectively. A known amount of MCDs-DOX conjugates (10 mg) was dispersed in 10 mL of PBS (pH 7.4 or pH 5.5) in a dialysis bag (MWCO: 2 kDa). The dialysis bag was placed in a 50 mL beaker containing the release medium, and the system was maintained at 37°C in a shaking water bath at 100 rpm to simulate physiological conditions. At predetermined time intervals, 1 mL of the release medium was withdrawn and replaced with an equal volume of fresh PBS to maintain constant volume. The released DOX concentration was measured using fluorescence spectroscopy (S2 Fig), with an excitation wavelength of 480 nm and an emission wavelength of 590 nm, corresponding to the characteristic fluorescence of DOX. The cumulative percentage of DOX released (% C) was calculated using the following formula:


$$\%\;C=\;\frac{Q_{t}}{Q_{0}}\times 100\mathrm{\backslash ]}$$ (1)

Where Q_t_ is the amount of DOX released at time ‘t’, Q_0_ is the initial amount of DOX loaded into the MCDs-DOX conjugates.

The encapsulation efficiency (EE) of DOX within the MCDs was determined by measuring the difference between the total amount of DOX used for the conjugation and the amount of free DOX not bound to the MCDs. To calculate EE, an aliquot of the MCDs-DOX conjugates (after purification) was dissolved in DMSO, and the free DOX was quantified by measuring its absorbance at 480 nm using a UV-Vis spectrophotometer.

The EE was calculated using the following formula:


$$EE(\%)=\frac{W_{encapsulated}}{W_{total}}\times 100$$ (2)

Where W_encapsulated_ is the amount of DOX encapsulated within the MCDs-DOX conjugates (calculated by subtracting the free DOX from the total amount of DOX initially added), and W_total_ is the total amount of DOX used for the conjugation process.

## 3. Results and discussions

CDs derived from Musa paradisiaca (MCDs) were synthesized via a simple, eco-friendly hydrothermal process. Dried and powdered banana peels were mixed with water and heated at 50–60 °C to extract bioactive compounds. The suspension was then subjected to hydrothermal treatment at 180 °C for 8 hours, enabling carbonization and formation of nitrogen-doped CDs. This method leverages an abundant agricultural waste for sustainable nanomaterial production. To optimize the hydrothermal synthesis of MCDs, various reaction temperatures (160 °C, 180 °C, 200 °C, and 220 °C) and durations (4, 8, and 12 hours) were systematically studied. It was observed that 200 °C for 8 hours produced MCDs with the most desirable properties, including uniform particle size, strong photoluminescence, and high antioxidant activity. At lower temperatures or shorter durations, the carbonization was incomplete, leading to reduced fluorescence, while higher temperatures resulted in particle aggregation.

**Fig 1:**
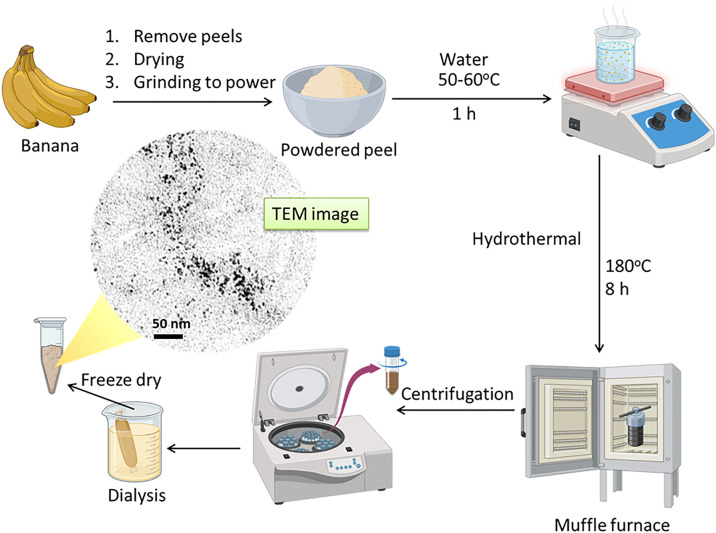
Diagrammatic representation of the synthesis of MCDs from *Musa paradisiaca* peels.

Hydrothermal synthesis is a prevalent method for producing carbon nanomaterials, since it facilitates the generation of homogeneous particles under regulated circumstances. Post-hydrothermal treatment, the resultant solution undergoes centrifugation to exclude bigger particles and unreacted residues, yielding a homogeneous suspension of CDs. Dialysis is conducted to remove tiny molecular contaminants and unreacted precursors, hence enhancing the sample’s purity. The filtered solution is then freeze-dried to provide MCDs in powder form, as seen in the bottom-left area of Fig 1. This phase guarantees the product’s stability and enhances its storage and handling. The TEM examination shown in the picture illustrates the morphological features of the synthesized MCDs. The TEM picture reveals that the MCDs possess a spherical morphology with an average diameter of around 50 nm. The consistency of the particle size distribution and the lack of substantial agglomeration underscore the effectiveness of the synthesis technique. The nanoscale size of the MCDs, together with their uniform shape, is crucial for their functional features, including fluorescence, antioxidant activity, and catalytic performance. The use of *Musa paradisiaca* peels offers a sustainable precursor while imparting inherent bioactive qualities to the produced CDs, attributable to the natural phytochemicals present in the peels. These phytochemicals may affect the surface properties of the MCDs, endowing them with distinct chemical and optical attributes. The integration of green and waste valorization strategies in nanomaterial synthesis tackles significant environmental issues, making this technique a viable alternative to traditional, resource-demanding technologies. The outlined synthesis technique efficiently converts agricultural waste into useful carbon nanoparticles. This method’s simplicity, scalability, and minimum environmental effect indicate its appropriateness for extensive applications in bioimaging, medication administration, and environmental cleanup. Future research will concentrate on the comprehensive characterization of surface chemistry and the investigation of the functional features of these MCDs in specific applications.

### 3.1 Optical and structural analysis

**Fig 2:**
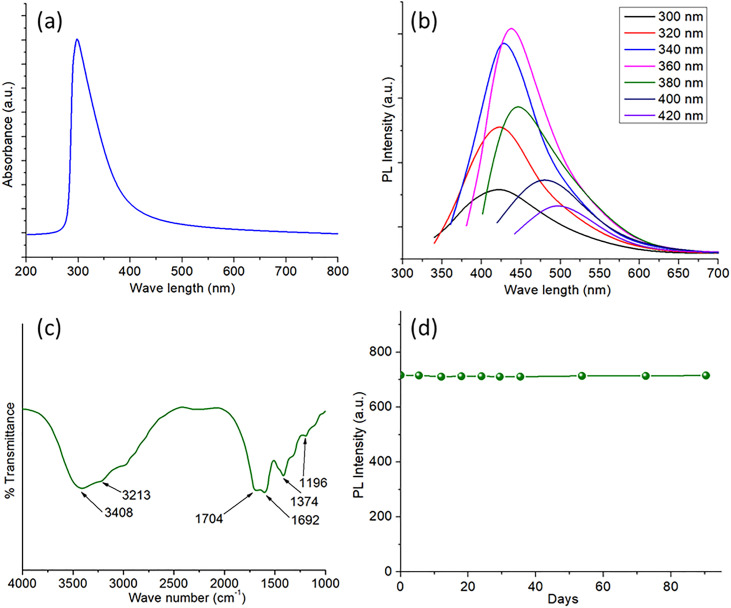
(a) UV-vis spectrum of MCDs (b) excitation dependent PL spectra of MCDs (c) FTIR spectrum of MCDs (d) PL intensities of MCDs for 90 days showing its stability.

Fig 2 fully presents the optical, structural, and stability aspects of the MCDs, including UV-Vis absorption, PL, Fourier-transform infrared spectroscopy (FTIR), and long-term photostability analyses. The UV-vis spectra of the MCDs, seen in Fig 2a, display a significant absorption peak at 280 nm, indicative of the π–π* transitions of aromatic C = C bonds [40]. The shoulder seen at this peak may be ascribed to n–π* transitions of C = O or other oxygen-containing functional groups present on the MCDs’ surface. This signifies the existence of conjugated domains inside the structure of the CDs, a defining attribute of fluorescent carbon nanomaterials. The PL properties of the MCDs, illustrated in Fig 2b, display excitation-dependent emission characteristics. The maximum PL intensity is observed at an excitation wavelength of 340 nm, with the emission peak about 450 nm, signifying a bright blue fluorescence. As the excitation wavelength increases from 300 nm to 420 nm, the emission peak undergoes a red shift accompanied by a gradual decrease in intensity. The excitation-dependent emission properties of CDs arise from the heterogeneous distribution of surface states, particle size, and the presence of several emissive sites on the MCDs. The functional groups on the MCDs were examined using FTIR spectroscopy, as seen in Fig 2c. The extensive absorption bands at 3408 cm ⁻ ¹ and 3213 cm ⁻ ¹ are attributed to O–H and N–H stretching vibrations, indicating the existence of hydroxyl and amine groups on the surface. The peaks at 1704 cm ⁻ ¹ and 1692 cm ⁻ ¹ are attributed to the stretching vibrations of C = O groups, whilst the band at 1374 cm ⁻ ¹ is associated with C–N stretching. The signal at 1196 cm ⁻ ¹ indicates C–O stretching vibrations, hence confirming the existence of oxygen-containing functional groups. The surface functions significantly influence the optical characteristics and solubility of the MCDs and may potentially improve their biocompatibility for prospective biological applications. The stability of the MCDs was assessed by tracking their PL intensity over a duration of 90 days, as seen in Fig 2d. The little fluctuation in PL intensity throughout this interval underscores the remarkable photostability of the MCDs. This stability is vital for bioimaging and optoelectronic applications, where reliable fluorescence emission over time is important. Moreover, the enduring stability of these MCDs renders them appropriate for applications necessitating extended exposure to light or environmental conditions.

**Fig 3:**
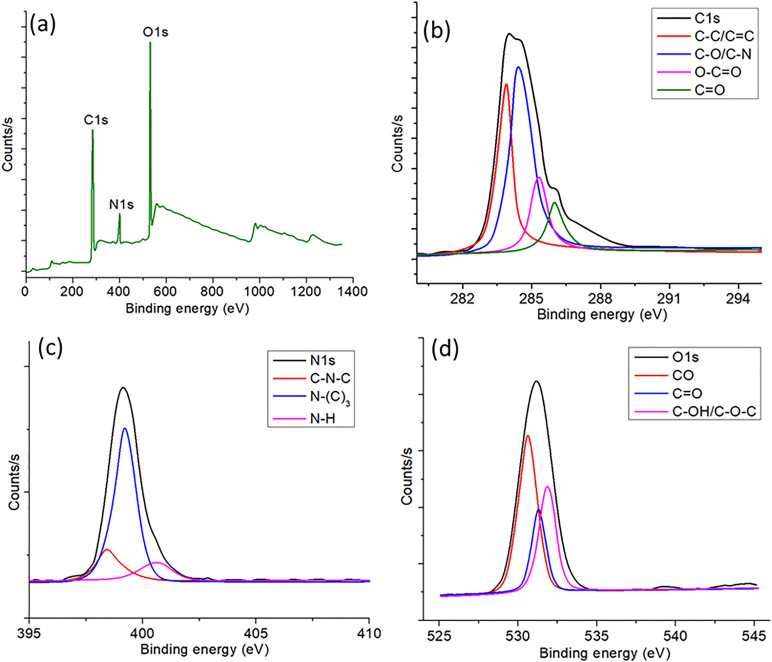
(a) XPS survey of the synthesized MCDs. High-resolution spectra of (b) C1s, (c) N1s, and (d) O1s scans for the synthesized MCDs.

The surface chemical composition and bonding states of the manufactured MCDs were examined by XPS, as seen in Fig 3. The XPS survey spectrum and high-resolution deconvoluted spectra of C1s, N1s, and O1s provide essential insights into the elemental composition and surface functions of the MCDs. The broad XPS spectrum in Fig 3a verifies the existence of carbon (C1s), oxygen (O1s), and nitrogen (N1s) in the MCDs, with corresponding binding energy peaks detected at about 284 eV, 532 eV, and 399 eV, respectively. The predominance of carbon and oxygen components indicates the carbonaceous characteristics of the MCDs and their oxygen-rich functional groups [41], but the presence of nitrogen implies effective integration of nitrogen dopants, possibly derived from the precursor or synthesis method. The integration of nitrogen is essential for optimizing the electrical configuration and improving the fluorescence characteristics of the MCDs. The high-resolution deconvolution of the C1s peak (Fig 3b) identifies four separate peaks with binding energies of about 284.8 eV, 285.6 eV, 287.2 eV, and 288.4 eV [42]. These peaks correspond to C–C/C–H (sp² carbon framework), C–O/C–N, C = O, and O–C = O bonds, respectively. The existence of C–O and O–C = O groups signifies that the MCDs possess abundant oxygen-rich surface functionalities, which are essential for enhancing their water dispersibility and fluorescence emission. The C–N peak indicates effective nitrogen doping, which enhances the quantum yield and modifies the electrical characteristics of the MCDs. The deconvoluted N1s spectra (Fig 3c) exhibit three distinct peaks at approximately 398.6 eV, 399.8 eV, and 401.3 eV, corresponding to pyridinic N (C = N–C), pyrrolic N (N–C₃), and graphitic N (N–H), respectively. The pyridinic and pyrrolic nitrogen species are associated with nitrogen atoms situated at the edges of the carbon framework or within heterocyclic structures, whereas graphitic nitrogen refers to nitrogen atoms that replace carbon atoms in the graphitic lattice (19). The existence of these nitrogen species indicates the effective integration of nitrogen into the carbon matrix, anticipated to improve the optical characteristics and catalytic efficiency of the MCDs. The O1s spectra (Fig 3d) are deconvoluted into three peaks at around 531.2 eV, 532.5 eV, and 533.6 eV, which correspond to C = O, C–O–C, and C–OH groups, respectively. The presence of oxygen-containing functional groups enhances the hydrophilicity of the MCDs and is crucial for their interaction with biomolecules and other substrates, rendering the MCDs appropriate for biosensing and bioimaging applications. The XPS analysis verifies that the produced MCDs exhibit a diverse surface chemistry defined by oxygen- and nitrogen-containing functional groups. These groups enhance the MCDs’ superior water dispersibility, biocompatibility, and adjustable fluorescence characteristics. The presence of C = O, C–N, and O–C = O groups on the surface offers active sites for further functionalization or conjugation with target molecules, hence augmenting the use of these MCDs in environmental, biological, and sensing applications.

**Fig 4:**
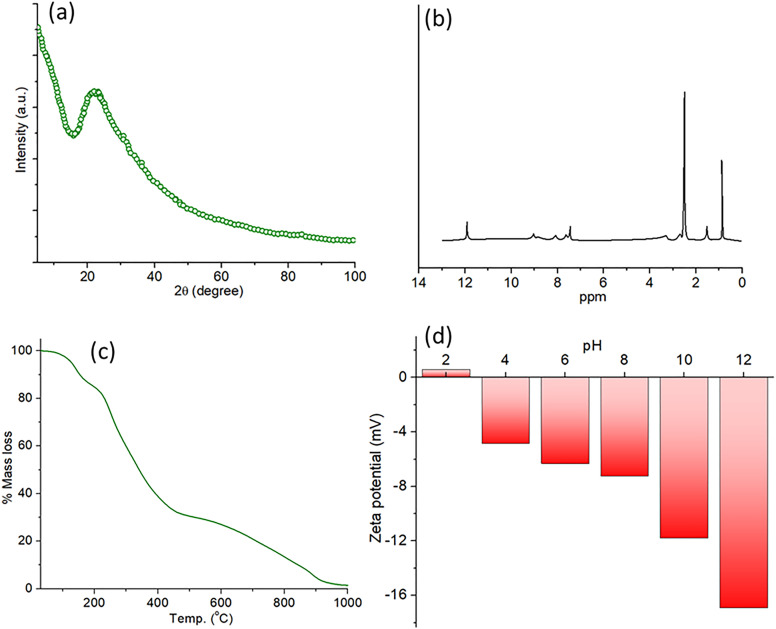
(a) XRD of MCDs (b) ^1^H NMR of MCDs (c) TGA of MCDs showing its thermal stability (d) pH-dependent zeta potentials of the MCDs in aqueous media.

Fig 4 offers a detailed characterisation of the molecular CDs (MCDs), emphasizing their structural, chemical, thermal, and surface attributes. Fig 4(a) depicts the X-ray diffraction (XRD) pattern of MCDs, with a large peak centered at 22°, which signifies their amorphous nature with graphitic domains, a trait often seen in carbon-based nanomaterials. The peak at ~22° (2θ) corresponds to the (002) plane of graphite, indicating stacked graphene layers. Its broadening suggests disordered or amorphous graphitic domains, consistent with turbostratic stacking or small crystallite size, as observed in amorphous carbon materials [43]. Fig 4(b) displays the proton nuclear magnetic resonance (^1^H NMR) spectrum of MCDs, revealing distinct peaks in the 6–8 ppm range indicative of aromatic protons, while peaks between 1–4 ppm imply the existence of aliphatic groups, thereby confirming a heterogeneous functional group composition on the MCD surfaces. The ^1^H NMR spectrum of the CDs exhibits a peak at 12 ppm, characteristic of carboxylic acid protons (–COOH), confirming surface oxidation during hydrothermal synthesis. Peaks in the 1–4 ppm region arise from oxygenated aliphatic moieties (e.g., –CH_2_–COOH, –CH_2_ –OH) and residual biomass fragments, reflecting the banana peel precursor’s polysaccharide-derived structure [44].

Fig 4(c) illustrates the thermal stability of MCDs using thermogravimetric analysis (TGA), indicating a progressive weight loss as temperature rises. Approximately 40% mass loss transpires below 300 °C, due to the elimination of surface functional groups and adsorbed moisture, followed by a gradual deterioration at higher temperatures, signifying the resilient carbonaceous core structure. Fig 4(d) depicts the pH-dependent zeta potential measurements of MCDs in aqueous conditions, indicating a transition from positive to negative values with increasing pH. In acidic pH (2–6), the zeta potential is positive to neutral, but in alkaline circumstances (pH 8–12), it becomes progressively negative, reaching a maximum of roughly −12 mV, indicative of the ionization of functional groups like carboxyl and hydroxyl on the MCD surface. Collectively, our findings confirm the structural stability, surface chemistry, and pH-responsive characteristics of MCDs, which are essential for their prospective applications in many domains like sensing, catalysis, and biological research.

### 3.2 Antioxidant properties

CDs represent a category of nanomaterials that have attracted considerable interest owing to their distinctive characteristics, including exceptional stability, minimal toxicity, and robust photoluminescence. These nanomaterials have demonstrated significant potential across a range of applications, such as bioimaging, drug delivery, and environmental sensing [45–47]. Besides their optical characteristics, CDs are recognized for their antioxidant qualities, rendering them appropriate for both medical and environmental protection applications [48]. Antioxidants eliminate reactive oxygen species (ROS) and free radicals, therefore diminishing oxidative stress in biological systems. The DPPH (2,2-diphenyl-1-picrylhydrazyl) and ABTS (2,2’-azino-bis(3-ethylbenzothiazoline-6-sulfonic acid)) assays serve as standard methods for assessing the antioxidant activity of substances such as CDs. The DPPH assay quantifies the capacity of an agent to scavenge DPPH radicals, which exhibit a distinct deep purple coloration. The decrease of DPPH radicals by antioxidants leads to a color alteration, signifying the degree of radical neutralization. The ABTS test similarly employs ABTS radicals, characterized by their green color, and quantifies their neutralization by antioxidants by the measurement of absorbance variation. Both assays serve as important tools for assessing the scavenging efficiency of CDs against various types of free radicals. They offer insights into the potential of CDs as potent antioxidants across a range of applications, including biomedical and environmental contexts.

**Fig 5:**
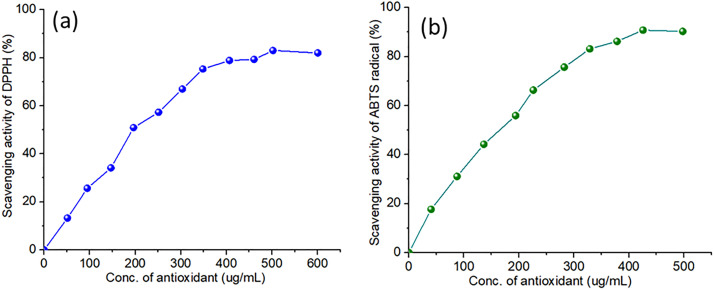
(a) Antioxidant activity of DPPH against CDs (b) Antioxidant activity of ABTS radical against CDs.

Fig 5a illustrates the scavenging activity of DPPH radicals by CDs at varying concentrations. With an increase in the concentration of CDs, there is a corresponding increase in scavenging activity, indicating that CDs display a dose-dependent antioxidant impact. At elevated concentrations (approximately 500 μg/mL), the scavenging activity nears 80%, demonstrating that CDs exhibit significant efficacy in neutralizing DPPH radicals. Fig 5b demonstrates the scavenging activity of ABTS radicals by CDs at different concentrations. The findings indicate a dose-dependent increase in scavenging activity, reaching around 90% at concentrations close to 500 μg/mL of CDs. This provides additional confirmation of the robust antioxidant activity exhibited by CDs, which presents a somewhat different profile in comparison to DPPH.

The antioxidant activity of CDs is mainly due to their surface chemistry and distinctive electronic properties. CDs generally contain functional groups including -OH, -NH2, and –COOH groups, which can donate electrons or hydrogen atoms and eliminate free radicals. The surface groups are essential for the interaction with reactive species such as DPPH and ABTS radicals. The elevated surface area-to-volume ratio of CDs increases their ability to scavenge radicals by offering a greater number of active sites for interaction. The conjugated π-electron system in the carbon core contributes to antioxidant properties by facilitating electron delocalization, aiding in the stabilization of radical species. The integration of surface functionalization with electronic structure enables CDs to proficiently neutralize reactive oxygen and nitrogen species, thereby establishing their role as effective antioxidants (12).

### 3.3 Drug loading and release

**Fig 6:**
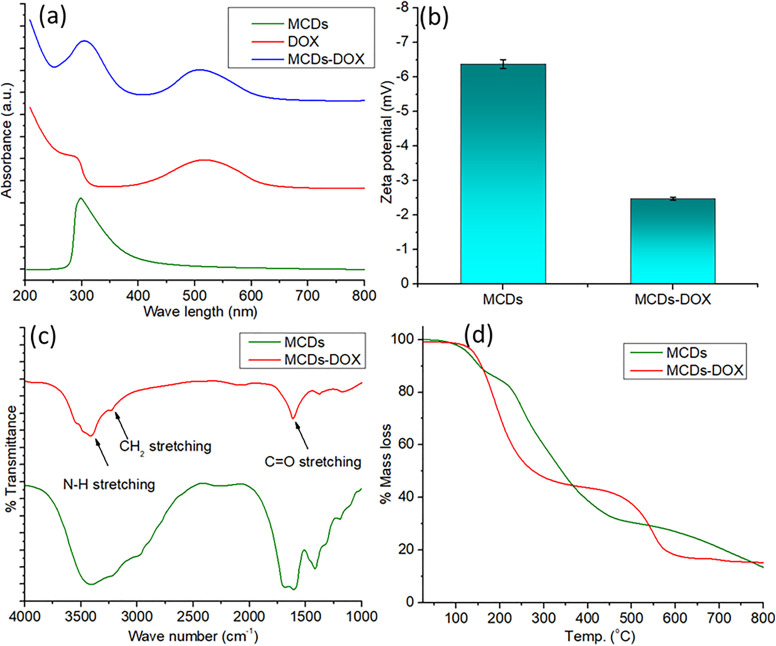
(a) UV-Vis spectra of pure MCDs, pure DOX and MCDs-DOX conjugate (b) surface charge analysis of MCDs and MCDs-DOX conjugate by zeta potential (c) FTIR spectra of MCDs and MCDs-DOX conjugate (d) TGA of MCDs and MCDs-DOX nanocomposite.

The effective loading of DOX onto MCDs was validated using several characterisation approaches, as seen in Fig 6. The UV-Vis spectra elucidate the interaction between MCDs and DOX (Fig 6a). The pure MCDs have distinct absorbance peaks at 280 nm, indicative of π–π* transitions in aromatic rings. Conversely, pure DOX has significant absorption around 480 nm, reflecting its molecular composition. The MCDs-DOX conjugate exhibits a dual peak, with increased absorbance at 480 nm, validating the effective conjugation of DOX to the MCDs. The spectral overlap signifies the stable binding of the medication via physical or chemical interactions. The zeta potential measurements, as shown in Fig 6b indicate changes in surface charge after DOX conjugation. MCDs display a zeta potential of roughly −7 mV, but the MCDs-DOX conjugate has a diminished zeta potential of about −2 mV. The transition to a reduced negative charge indicates that the electrostatic characteristics of the MCDs surface have been altered by the binding of DOX molecules, hence affirming effective drug loading. The FTIR spectra (Fig 6c) elucidate the functional groups involved in drug conjugation. The spectra of pure MCDs have a wide N-H stretching peak about 3300 cm ⁻ ¹, along with distinct peaks associated with C = O and CH- stretching. Following DOX conjugation, the spectra of MCDs-DOX exhibit a notable amplification in C = O stretching vibrations about 1700 cm ⁻ ¹, signifying interactions between the amine or hydroxyl groups of DOX and the surface functional groups of MCDs. Pure DOX FTIR (S1 Fig) is also confirms the peaks after conjugation. This verifies the chemical bonding or robust physical contact between MCDs and DOX. The thermal stability of MCDs and MCDs-DOX was assessed to measure drug loading, as shown in Fig 6d. The TGA curves indicate a greater mass loss in MCDs-DOX relative to pure MCDs. In MCDs, a progressive weight reduction of about 40% transpires up to 700°C, mostly resulting from the degradation of carbonaceous components. Conversely, the MCDs-DOX conjugate shows a weight loss of roughly 50%, resulting from the disintegration of DOX molecules in conjunction with MCDs. This disparity indicates the effective integration of DOX, since the increased weight loss correlates with the drug’s presence.

**Fig 7:**
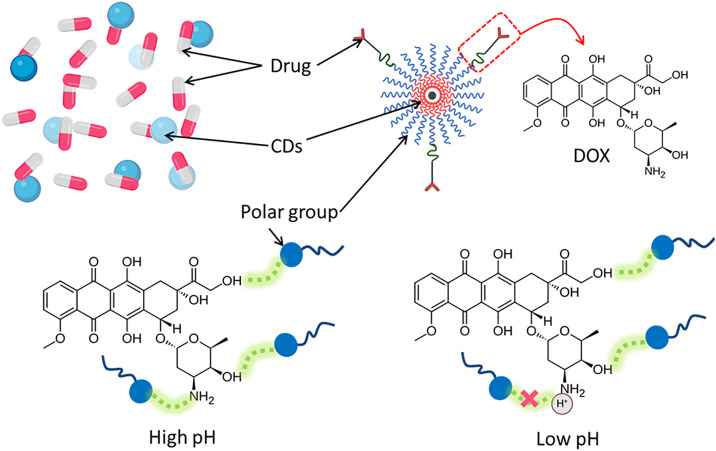
Schematic illustration of CDs-drug interaction at low and high pH.

Fig 7 illustrates the interaction between MCDs and the anticancer agent DOX at varying pH levels, highlighting the pH-sensitive properties of the drug-conjugate system. MCDs, distinguished by their many polar functional groups, perform as an effective nanocarrier platform for drug binding through electrostatic, hydrogen bonding, and π–π stacking interactions. At high pH, the deprotonation of the hydroxyl and amino groups on DOX enhances its interaction with the negatively charged MCDs. This leads to a resilient attachment enabled by strong electrostatic and hydrogen-bonding interactions, ensuring efficient drug loading. Conversely, at low pH, characteristic of acidic tumor microenvironments, the amine group of DOX becomes protonated, reducing its affinity for the MCDs. Protonation reduces electrostatic interactions and disrupts hydrogen bonds, hence promoting the release of the drug from the cyclodextrins. This pH-sensitive drug release mechanism is especially beneficial for targeted administration in cancer treatment, since it minimizes drug leakage under normal physiological settings (neutral pH) while facilitating increased release in acidic environments typical of tumors. This intelligent drug delivery device not only showcases effective loading but also displays a responsive release profile influenced by pH fluctuations. The features of the MCDs-DOX nanocomposite render it an attractive option for pH-sensitive therapeutic applications, using tumor-specific acidity for selective and regulated drug release. The schematic highlights the potential of MCDs for site-specific drug delivery via meticulously engineered surface changes and interactions.

The release kinetics of DOX from CDs-DOX nanocomposites at varying pH levels is examined using three principal models: The Higuchi model, the Korsmeyer-Peppas model, and the Peppas-Sahlin model. These models elucidate the mechanisms of drug release, clarifying diffusion, polymer relaxation, and other physicochemical interactions.

The Higuchi model describes drug release based on diffusion from a homogenous matrix [49]. The equation is:


$$m_{Dt}=m_{De}+K_{H}t^{\frac{1}{2}}\mathrm{\backslash ]}$$ (3)

Where Q_t_ is the cumulative amount of drug released at time ‘t’, and k_H_ is the Higuchi release constant. This model assumes a Fickian diffusion mechanism, making it suitable for systems where drug release is dominated by diffusion through a matrix.

Korsmeyer-Peppas Model is a semi-empirical equation used for systems where drug release mechanisms are not purely Fickian [50]. The equation is:


$$F(D)=\frac{m_{Dt}}{m_{De}}=K_{KP}t^{n}\mathrm{\backslash ]}$$ (4)

Where Q_t_/Q_∞_ is the fractional release, k_KP_ is the release rate constant, and nnn is the release exponent, which indicates the type of release mechanism. For n < 0.5, the release is Fickian diffusion; for 0.5 < n < 1, it is anomalous (non-Fickian), and for n = 1, it is zero-order.

The Peppas-Sahlin model [51] combines Fickian diffusion and polymer relaxation effects. The equation is:


$$Q_{t}={\text{K}}_{\text{\{1}}\}{\text{t}}^{\text{m}}+K_{2}t^{2m}$$ (5)

Where K_1_ and K_2_ are constants related to Fickian and relaxation mechanisms, respectively, and mmm is the diffusional exponent. This model is particularly useful for systems with a combined contribution of these mechanisms.

**Fig 8:**
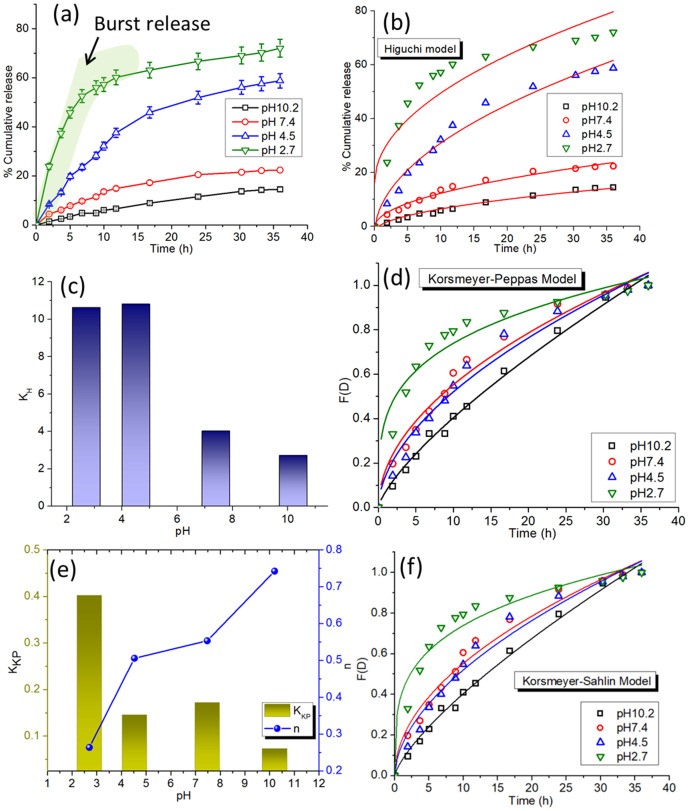
(a) Cumulative drug release study of CDs-DOX nanocomposites at various pH environments (b) Fittings of the release data in Higuchi model (c) Histogram of Higuchi constant values at different pHs (d) Fittings of the release data in Korsmeyer-Peppas model (e) Histogram of Peppas constant and release exponent at different pHs (f) Peppas-Sahlin model fittings of the release data.

The cumulative release profile (Fig 8a) demonstrates a distinct pH-dependent drug release pattern, marked by an initial rapid burst phase succeeded by a longer, sustained release. At pH 5.0, mimicking the acidic tumor microenvironment, more than 60% of DOX is released after 48 hours, but only 30% is released at pH 7.4, representative of neutral normal conditions. The enhanced release at acidic pH results from the protonation of DOX, which reduces its electrostatic and hydrogen-bonding interactions with MCDs, hence facilitating its diffusion. The Higuchi model fitting (Fig 8b) confirms a diffusion-dominated mechanism, evidenced by increased kH values at lower pH levels. The histogram of k_H_ values (Fig 8c) demonstrates a significant rise at pH 5.0, highlighting the impact of acidic circumstances on drug transport. The Korsmeyer-Peppas model fitting (Fig 8d) clarifies the integrated influence of diffusion and polymer relaxation in the release mechanism. The release exponent *n*, which ranges from 0.5 to 0.6, indicates an atypical (non-Fickian) mechanism in which both processes are involved. Fig 8e illustrates the pH dependence, revealing elevated k_KP_ and *n* n values at acidic pH, so confirming the enhanced release. Simultaneously, the Peppas-Sahlin model fitting (Fig 8f) elucidates the processes, indicating that K_1_ (Fickian contribution) predominates at neutral pH, but K_2_ (relaxation-controlled contribution) markedly escalates at pH 5.0. This signifies that polymer relaxation, governed by pH-sensitive interactions, is an essential characteristic in acidic environments. The drug release kinetics of MCDs-DOX nanocomposites exhibit notable pH sensitivity, with enhanced release in acidic environments due to the protonation of DOX, which hinders its interaction with MCDs. The initial release phase at pH 5.0, succeeded by a sustained release, aligns with the diffusion-controlled mechanism described by the Higuchi model, whereas the combined insights from the Korsmeyer-Peppas and Peppas-Sahlin models highlight the synergistic influences of diffusion and polymer relaxation. The results underscore the suitability of MCDs-DOX nanocomposites for targeted drug delivery in acidic tumor microenvironments, enhancing drug release and therapeutic efficacy. The enhanced release at acidic pH is attributed to the protonation of surface functional groups (such as –COOH and –NH_2_) on the MCDs, which increases hydrophilicity and weakens drug-MCD interactions, facilitating drug release under acidic conditions typical of tumor microenvironments or inflamed tissues.

### 3.4 Biocompatibility analysis

**Fig 9:**
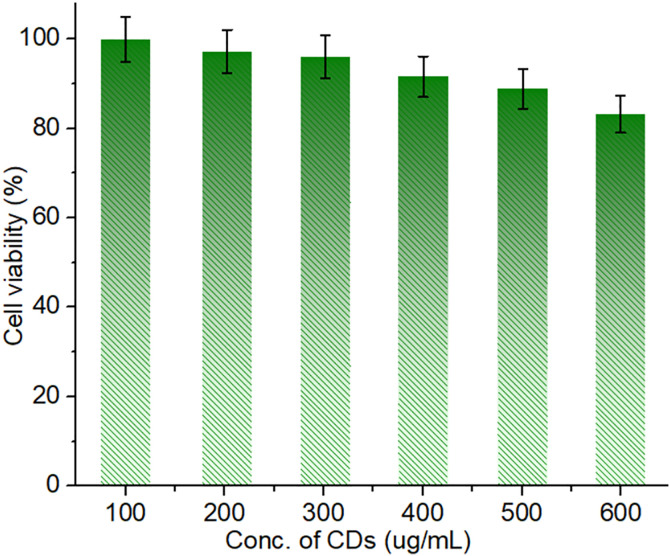
Biocompatibility test of the CDs against living cell lines.

The MTT assay findings shown in Fig 9 validate the remarkable biocompatibility of the MCDs when evaluated against the 3T3 fibroblast cell line within a concentration range of 100–600 µg/mL. The cell viability% % stays constantly elevated, with few indications of cytotoxicity even at the maximum measured dose. At 600 µg/mL, cell viability remains over 85%, indicating the nontoxic nature of the MCDs for cellular applications. The little decrease in cell viability seen with escalating doses is negligible and within acceptable parameters, confirming that the MCDs are non-toxic and compatible with biological systems. Moreover, the lack of notable cytotoxic effects indicates that these MCDs may allow extended exposure in cellular contexts without jeopardizing cell viability. The increased biocompatibility of the MCDs renders them very promising for biomedical applications, such as drug administration, bioimaging, and tissue engineering, where non-toxicity is essential. The findings robustly confirm the promise of these MCDs for clinical and therapeutic applications, highlighting their appropriateness for sophisticated biological uses.

Compared to conventional multi-step or template-based synthesis approaches, our single-step hydrothermal method offers a scalable, cost-effective, and environmentally benign route, using a renewable and abundant biomass precursor (Musa paradisiaca) without additional chemical dopants or surfactants. This minimizes chemical waste and energy consumption, making it suitable for green production at larger scales. Furthermore, the excellent biocompatibility, high antioxidant efficiency, and controlled drug release behavior of MCDs highlight their strong potential for clinical applications, particularly in oxidative stress-related therapies, targeted drug delivery, and bioimaging. These aspects enhance the translational relevance and novelty of our work.

## Conclusions

This work emphasizes the effective creation and characterization of multifunctional MCDs-DOX nanocomposites as a viable platform for drug delivery applications. The conjugation of DOX with MCDs was validated using UV-Vis spectroscopy, FTIR analysis, and zeta potential measurements, therefore confirming the effective loading of the medication onto the CDs. Drug release studies showed pH-responsive characteristics, with enhanced release in acidic conditions, simulating tumor microenvironments. This selective release profile benefits targeted cancer treatment by reducing systemic medication exposure and improving therapeutic effectiveness. The kinetics of drug release was meticulously assessed by mathematical models, such as the Higuchi, Korsmeyer-Peppas, and Peppas-Sahlin models, elucidating the processes driven by diffusion and polymer relaxation. The observations indicate a regulated release profile influenced by both Fickian diffusion and non-Fickian transport processes, which corresponds effectively with the demands of sophisticated drug delivery systems. Biocompatibility assessments via MTT assays validated the non-toxic nature of the MCDs, sustaining over 85% cell viability in 3T3 fibroblasts, even at high concentrations. This enhances the safety profile of the MCDs, underscoring their potential for prolonged biological application. The ability of MCDs to function as pH-sensitive nanocarriers makes them an ideal choice for delivering chemotherapeutic agents to acidic tumor microenvironments, hence reducing off-target effects. The findings suggest that the optical properties of the MCDs can be utilized for theranostic applications, combining drug delivery with imaging capabilities. This study emphasizes the versatility and biocompatibility of MCDs-DOX nanocomposites, establishing a robust basis for targeted drug administration. Future study will focus on in vivo evaluations and exploring the theranostic potential of these nanocomposites, enabling their advancement into clinical applications. The results presented herein significantly enhance the emerging field of carbon dot-based nanomedicine, demonstrating its potential as an advanced drug delivery system.

## Supporting information

S1 Fig FTIR spectrum of pure DOX. (TIF)

S2 Fig Standard curve of DOX fluorescence intensity as a function of concentration. (TIF)

## Notes

During the preparation of this work we are used “Quillbot” in order to refine the language. After using this tool, we have reviewed and edited the content as needed and take(s) full responsibility for the content of the publication.
