# Supplementary figures and images for: Musa Paradisiaca derived intrinsically heteroatom doped carbon dots as antioxidant and controlled drug release behavior
Source: PLoS One. 2025 Aug 14;20(8):e0329116. doi: 10.1371/journal.pone.0329116 (PMC12352835; doi:10.1371/journal.pone.0329116)

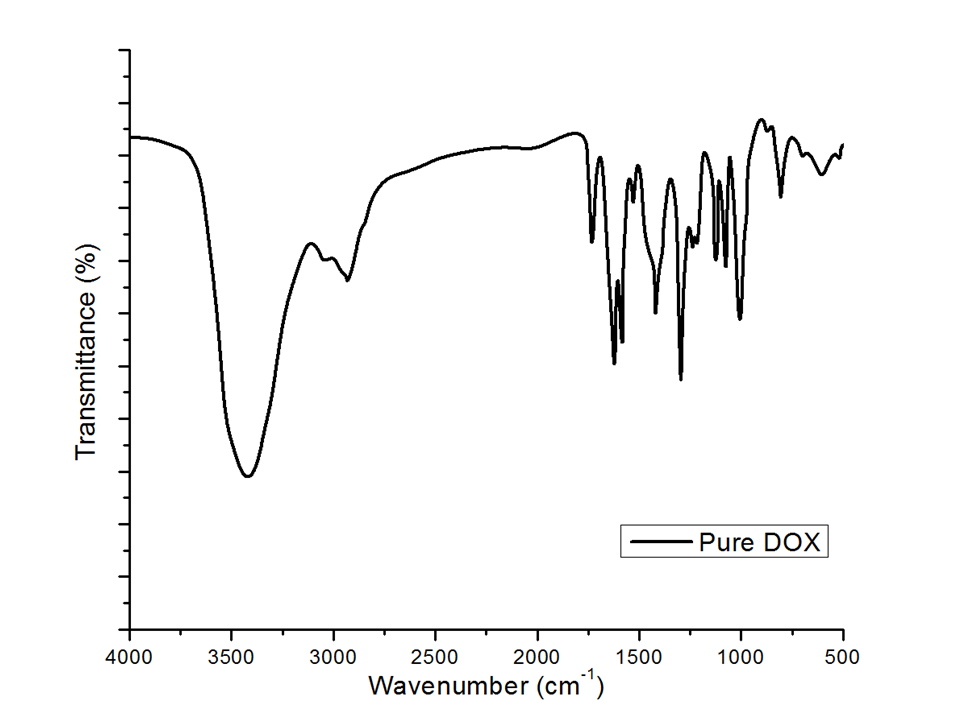

Supplement: S1 Fig — (TIF) [file pone.0329116.s001.tif]

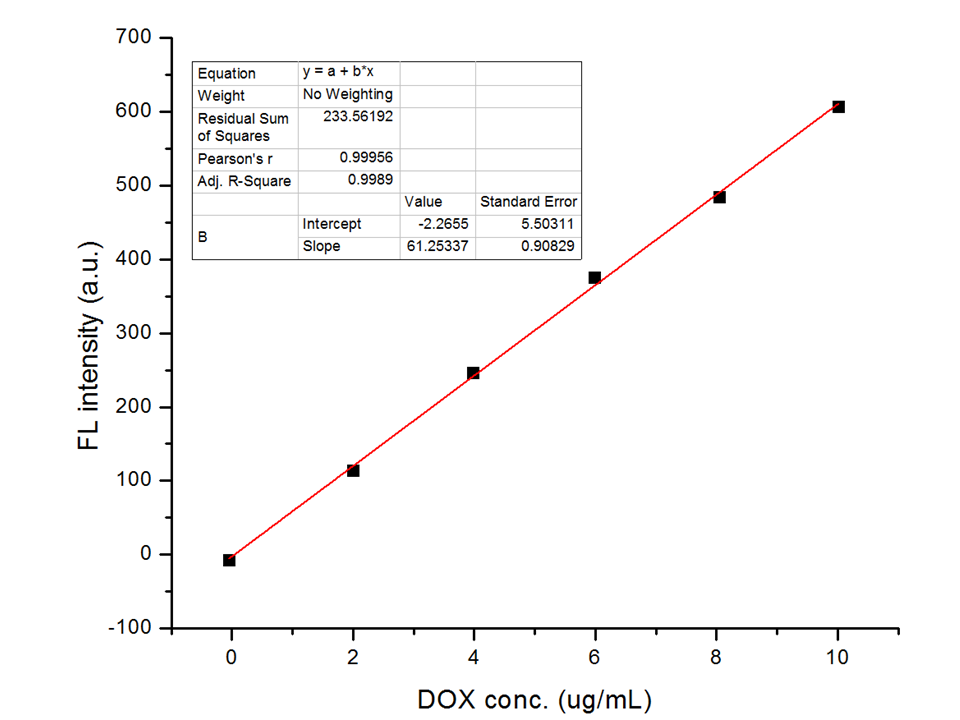

Supplement: S2 Fig — (TIF) [file pone.0329116.s002.tif]
